# Age Distribution, Comorbidities and Risk Factors for Thrombosis in Prader–Willi Syndrome

**DOI:** 10.3390/genes11010067

**Published:** 2020-01-07

**Authors:** Merlin G. Butler, Aderonke Oyetunji, Ann M. Manzardo

**Affiliations:** 1 Departments of Psychiatry & Behavioral Sciences and Pediatrics, University of Kansas Medical Center, Kansas City, KS 66160, USA; aoyetunji@kumc.edu (A.O.); amanzardo@kumc.edu (A.M.M.); 2 Department of Child Psychiatry, Truman Medical Centers, Kansas City, MO 64108, USA

**Keywords:** Prader–Willi syndrome, insurance health claims, thrombosis, pulmonary embolism, deep venous thrombosis, individuals with exogenous obesity, confirmatory ICD-9 diagnostic codes

## Abstract

Prader–Willi syndrome (PWS) is an imprinting disorder caused by lack of expression of the paternally inherited 15q11.2–q13 chromosome region. The risk of death from obesity-related complications can worsen with age, but survival trends are improving. Comorbidities and their complications such as thrombosis or blood clots and venous thromboembolism (VTE) are uncommon but reported in PWS. Two phases of analyses were conducted in our study: unadjusted and adjusted frequency with odds ratios and a regression analysis of risk factors. Individuals with PWS or non-PWS controls with exogenous obesity were identified by specific International Classification of Diseases (ICD)-9 diagnostic codes reported on more than one occasion to confirm the diagnosis of PWS or exogenous obesity in available national health claims insurance datasets. The overall average age or average age per age interval (0–17 year, 18–64 year, and 65 year+) and gender distribution in each population were similar in 3136 patients with PWS and 3945 non-PWS controls for comparison purposes, with exogenous obesity identified from two insurance health claims dataset sources (i.e., commercial and Medicare advantage or Medicaid). For example, 65.1% of the 3136 patients with PWS were less than 18 years old (subadults), 33.2% were 18–64 years old (adults), and 1.7% were 65 years or older. After adjusting for comorbidities that were identified with diagnostic codes, we found that commercially insured PWS individuals across all age cohorts were 2.55 times more likely to experience pulmonary embolism (PE) or deep vein thrombosis (DVT) than for obese controls (*p*-value: 0.013; confidence interval (CI): 1.22–5.32). Medicaid-insured individuals across all age cohorts with PWS were 0.85 times more likely to experience PE or DVT than obese controls (*p*-value: 0.60; CI: 0.46–1.56), with no indicated age difference. Age and gender were statistically significant predictors of VTEs, and they were independent of insurance coverage. There was an increase in occurrence of thrombotic events across all age cohorts within the PWS patient population when compared with their obese counterparts, regardless of insurance type.

## 1. Introduction

Prader–Willi syndrome (PWS) is a neurodevelopmental genomic imprinting disorder that results from the absence of paternally expressed imprinted genes at the 15q11.2–q13 chromosome region due to a paternal deletion of this region (60% of cases), maternal uniparental disomy 15 (36%), or an imprinting defect (4%) [1]. PWS is a rare genetic disorder that is associated with an incidence between 1 in 10,000–30,000 live births with a specific phenotype, and it is considered to be the most common known genetic cause of obesity [2,3,4]. Reports have suggested an important association between obesity and early death in adults with PWS [4]. Comorbidities that are commonly associated with obesity in PWS include respiratory problems (pulmonary embolism, respiratory failure, and pulmonary hypertension) and deep venous thrombosis [5,6,7,8,9].

An increased risk of venous thromboembolisms (VTEs) was recently reported in PWS patients by using Prader–Willi syndrome Association (USA) syndrome-specific database of deaths between 1973 and 2015 [5,9]. Seven percent of all deaths in the reported PWS survey commonly found in adulthood were attributable to pulmonary embolism, which represented only a quarter of the most common causes of death that were related to respiratory failure [5,6]. However, the reported deaths that resulted from respiratory failure could be secondary to undiagnosed pulmonary embolism and may vary by gender and/or age of patient [5]. Death in infancy/young childhood was found to be related to respiratory failure—more so than obesity-related factors. Pulmonary thromboembolism and obesity-related complications were more commonly found in adolescents and adults, with females more likely to suffer from obesity-related morbidity [5,10]. Blood clots, venous thrombosis, and/or pulmonary are recognized as contributors to morbidity and mortality in PWS in the United States.

As PWS is estimated to affect over 500,000 people worldwide and risk factors including obesity are on the rise, there is no doubt that the associated, marked obesity in PWS predisposes patients to thrombosis [4]. In addition, a venous thrombosis survey reported by Manzardo et al. [6] was carried out in more than 1000 individuals with PWS in the United States with an age range of 0–63 years. This survey revealed the presence of thrombosis in 3% of cases, with the incidence greater in females than males. Thirty-three of the identified 38 clotting events occurred at ages greater than 17 years [6]. The occurrence of these events was associated with a greater age and an increased weight or obesity, as determined by the body mass index (BMI). The types of reported events included thrombosis or specified blood clots, deep venous thrombosis (DVT), and pulmonary embolism (PE). DVTs showed the highest percentage at 21%. Obesity was the most common finding that was associated with PE and that was identified in the previous PWS survey of blood clots reported by Manzardo et al. [6].

To further investigate the associated risk factors that play a role in blood clots and thrombotic events in living patients with PWS, we utilized two sources of national healthcare insurance claims datasets based on International Classification of Diseases (ICD)-9 diagnosis codes [11]. We aimed to identify thrombotic events and their occurrence in a large number of patients with PWS and non-PWS obese controls across all age groups, and we used key variables to analyze independent predictors/risk factors.

## 2. Materials and Methods

We used insurance health claims data from the MarketScan Research Databases for the years 2004–2014 as a source of data to carry out a preliminary record search of primary thrombosis and related blood clotting events in patients with Prader–Willi syndrome. The patients were identified from commercial or Medicare Advantage and Medicaid insurance groups. Individuals with exogenous obesity were also included as non-PWS controls as a comparison group. The Market Scan Research Databases contain de-identified health insurance enrollment information and fully-adjudicated claims data for all medical services and prescribed medications. As an example of the number of claim reports, approximately six million Medicaid enrollees from multiple states had more than 500 million claim records available for analysis [11]. The dataset provides a national cross-sectional and longitudinal view of demographics and enrollment among others in the United States.

Our study design applied a coding methodology and analysis plans by searching diagnostic ICD-9, Diagnosis Related Group (DRG), and Current Procedural Terminology (CPT) codes for key variables in the health insurance claims. The analyses focused on the occurrence of primary thrombosis events in 3136 patients with PWS and a non-PWS cohort with exogeneous obesity that consisted of 3945 individuals. These patients were identified by recorded ICD-9 codes (PWS, ICD-9 code 759.81; exogenous obesity, ICD-9 code 278.44) found on more than one occasion to confirm the diagnosis in both cohorts for inclusion.

Primary analyses in Phase I used contingency two-factor tables and the Chi-square test. Event rates were given as a percentage of patients that did/did not have a thrombosis or blood clotting code during their entire length of continuous enrollment over a ten-year period from 2004 to 2014. Odds ratios (ORs) with confidence intervals (CI) were calculated by using a logistic regression model with an offset term to adjust for differing lengths of continuous enrollment and follow-up between the two subject cohorts; these ORs are presented in terms of relative risk (PWS compared to non-PWS obese controls). The odds ratios were used to represent the occurrences of primary thrombosis events in the PWS versus non-PWS obese control populations that were identified in these health claims datasets. 

Phase I quantified the occurrence of thrombosis events (did/did not have event) and calculated odds ratios though five prioritized analyses:

Analysis 1: PE and DVT codes
Analysis 2: PE and DVT and PWS confirmatory codes
Analysis 3: PE and DVT and other venous thrombosis codes
Analysis 4: PE and DVT and other venous thrombosis and PWS confirmatory codes
Analysis 5: Arterial thrombosis codes

**Figure 1:**
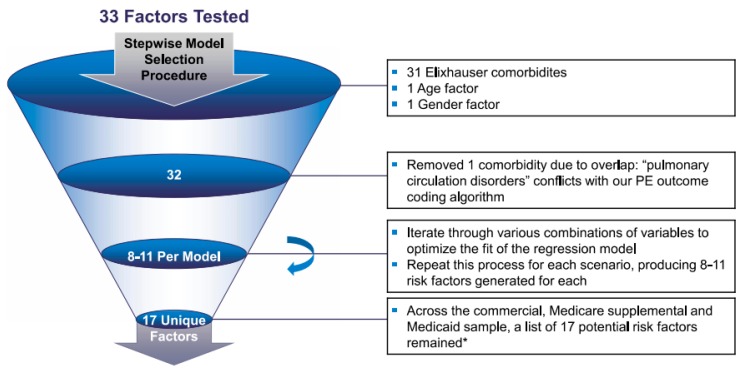
Iterative statistical stepwise approach to determine the risk factors that contributed significantly to thrombosis events.

Baseline comorbidity adjustment is considered an important component of research related to health services and clinical prognosis. When adjusting for comorbidities, investigators may consider comorbidities either individually or through the use of summary measures such as the Elixhauser comorbidity score, which is derived with regression estimates. A modified Elixhauser-adjusted method was used in this study, as previously described [12,13]. The Elixhauser- adjusted occurrences were primary thrombosis events and ORs that were calculated by using logistic regression models. Logistic regression models were adjusted for the 31 comorbidities contained in the Elixhauser comorbidities index. In Phase II, risk factors for thrombotic events were identified based on the Elixhauser comorbidities index by using a stepwise statistical selection approach to find a parsimonious model that incorporated 31 chronic diseases (which are discussed later) identified by ICD-9-CM codes (as well as age and gender as additional risk factors) deemed as comorbidities associated with thrombosis events in PWS [12,13] (see Figure 1). With significant risk factors/comorbidities identified as major contributors to primary thrombosis events, an unadjusted analysis of primary thrombosis events or blood clots was carried out in PWS patients relative to their obese non-PWS counterparts.

## 3. Results

**Figure 2:**
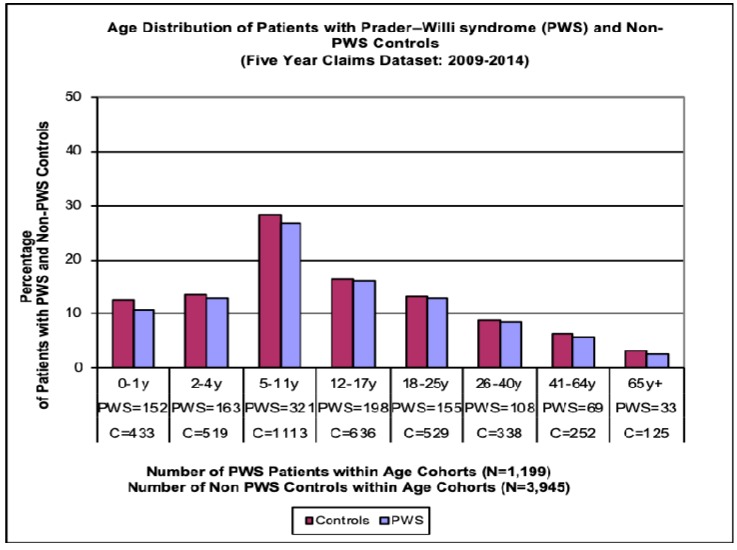
Age distribution bar graph of patients with Prader–Willi syndrome (PWS) and non-PWS obese controls.

A total of 1821 patients with PWS were available for analysis from one of the groups of subjects with commercial or Medicare Advantage insurance coverage, of which 51% were males and 49% were females. In addition, 1315 patients with PWS had a second form of insurance coverage (e.g., Medicaid), of which 54% were males and 46% were females. Thus, a total number of 3136 PWS patients were studied with 52.5% males and 47.5% females (see Table 1). A total of 3945 non-PWS controls with exogenous obesity were identified using the two insurance coverage types, and they were selected to represent the average age and distribution with gender ratio seen in the PWS population for comparison purposes. When combined, a total number of 2042 (65.1%) patients with PWS had an age range of 0–17 years, 1040 (33.2%) patients were between the ages of 18 and 64 years, and 52 (1.7%) patients with PWS were 65 years and older. Furthermore, this health claims dataset was used to study the medical costs in PWS that were reported by Shoffstall et al. [10], who found higher medical costs in a five year subset (2009–2014) for PWS in comparison to non-PWS obese patients who were selected for comparison with a similar gender ratio and age distribution seen in three age cohorts [11]. Additionally, the age for the 1199 PWS subjects and 3945 non-PWS obese subjects were further grouped into eight categories beginning with 0–3 years, 2–4 years, 5–11 years, 12–17 years, 18–25 years, 26–40 years, 41–64 years and 65 years and older (see Figure 2).

**Figure 3:**
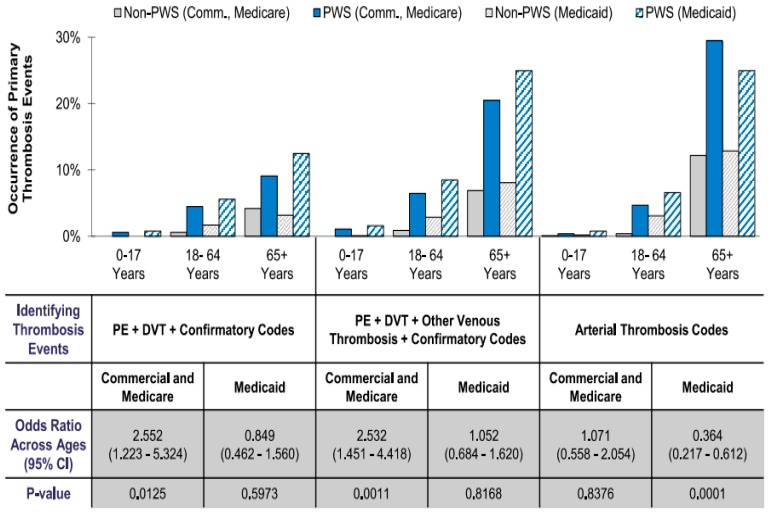
Adjusted primary thrombosis events of patients with Prader–Willi syndrome (PWS) in comparison to non-PWS obese patients across all age cohorts, insurances, and analyses.

Individuals with PWS experienced a higher overall frequency of thrombosis events than their similarly aged and gender-matched non-PWS obese counterparts across all analyses unadjusted for comorbidities. An addition of confirmatory codes to unadjusted thrombosis events caused a reduction in the total number of identified events, but the frequency of the events still trended higher across all age groups for all types of insurance coverage (see Figure 3).

**Figure 4:**
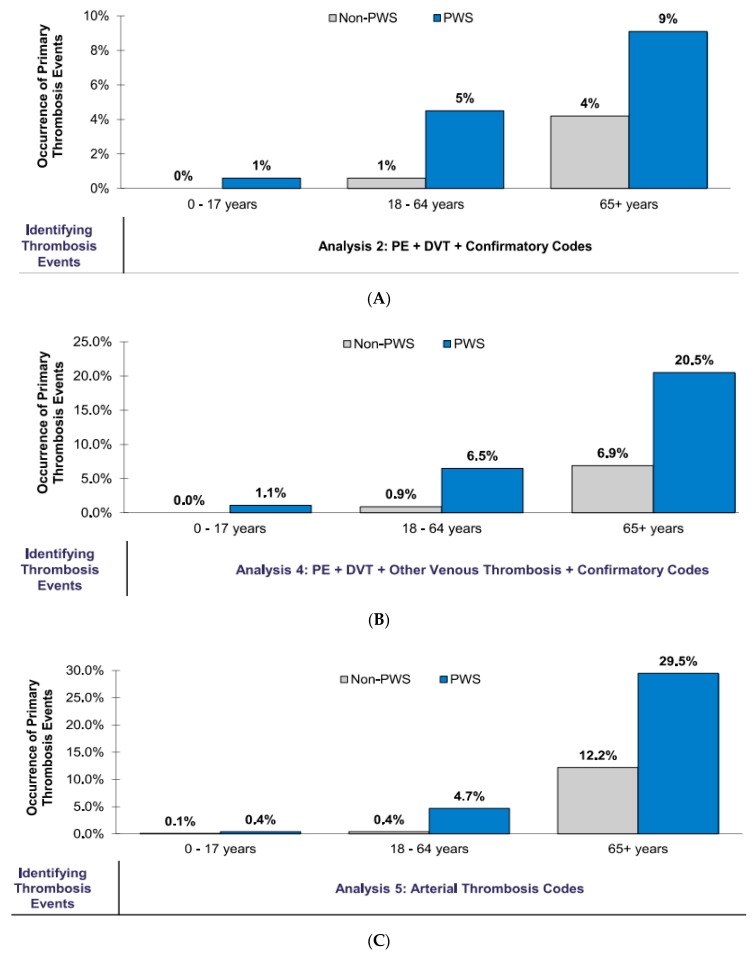
Primary thrombosis events in patients with Prader–Willi syndrome and non-PWS obese controls on commercial/Medicare coverage across all age cohorts with confirmatory codes in Analysis 2 (**A**), Analysis 4 (**B**) and Analysis 5 (**C**).

Overall, there were more occurrences across all age cohorts for PE and DVT with confirmatory codes (Analysis 2); other occurrences included venous thrombosis (Analysis 4) and arterial thrombosis (Analysis 5) (see Figure 4A–C). Commercially insured individuals with PWS were also 4.98 times more likely to experience PE or DVT events than the obese controls (*p*-value < 0.0001). Meanwhile, 1.8% of the individuals experienced PE or DVT events compared to 0.3% for their similar non-PWS aged and gender-matched obese counterparts. For PE, DVT, and “other” venous thrombotic codes, 2.9% of the PWS individuals and 0.5% of the non-PWS individuals experienced thrombosis events with odds ratio of 5.13 (*p*-value < 0.0001). Arterial events (i.e., stroke and heart attacks) occurred in 2.2% of the PWS individuals and 0.5% of the non-PWS individuals; OR = 3.88 (*p*-value < 0.0001) (see Table 2A).

In Phase 2, stepwise logistic regression identified 17 unique risk factors for PE or DVT out of the 31 chronic diseases modeled. The potential risk factors that were identified were, in alphabetical order, age cohort 18–64 (vs. 0–17), age cohort 65+ (vs. 0–17), AIDS/HIV, cardiac arrhythmias, chronic pulmonary disease, coagulopathy, congestive heart failure, deficiency anemia, diabetes uncomplicated, hypertension complicated, hypertension uncomplicated, male gender (vs. female), metastatic cancer, obesity, psychosis, rheumatoid arthritis/collagen, and solid tumor without metastasis (see Table 3).

Medicaid-insured individuals with PWS were 1.95 times more likely to experience PE or DVT events than the non-PWS obese controls (*p* = 0.0075), and 3.0% of these individuals experienced PE or DVT events compared to 0.7% of their similar non-PWS aged and gender-matched obese subjects. Including PE, DVT, and “other” venous thrombotic event codes, 4.8% of the PWS individuals and 1.3% of the non-PWS obese individuals experienced thrombosis events; OR = 1.88 (*p*-value = 0.0012).

Arterial events (i.e., stroke and heart attacks), occurred in 3.5% and 1.5% of the PWS and non-PWS cohorts, respectively. This difference was not statistically significant after controlling for differing lengths of enrollment (see Table 2B). However, this difference was statistically significant across all analyses for commercial/Medicare coverage, but it was not statistically significant for arterial thrombosis for Medicaid coverage (see Table 2A).

## 4. Discussion

Patients with PWS continued to show a greater likelihood of experiencing thrombosis events than their similarly gender-matched obese counterparts across all age cohorts, regardless of insurance type after adjusting for comorbidities by evaluating PWS-specific contributions to thrombosis or blood clot events and by removing potentially confounding effects from comorbidities (see Figure 4). The inclusion of confirmatory codes to identify PE and DVT events provided a clearer picture of disparity across ages. In our study, all patients were identified and grouped into two insurance health claims datasets (commercial/Medicare and Medicaid). Patients with PWS who had commercial/Medicare coverage showed a higher occurrence of PE and DVT events than their similar aged and gender-matched counterparts across all age cohorts. Both PWS patients and obese controls in the 65+ year age cohort experienced the highest amount of PE and DVT events. With the inclusion of confirmatory PWS diagnostic codes, the PE and DVT events still had a higher occurrence in PWS patients than their obese counterparts (see Figure 3). Even though the number of events was lower in the PWS population, though not for the controls in the 18–64-year age cohort, the PWS patients in the 65+ year age cohort experienced a higher occurrence of events compared with their obese counterparts.

With other venous thrombotic events, PWS patients with commercial/Medicare coverage still had a higher occurrence of venous thrombotic events than their similarly-matched obese counterparts across all age cohorts despite the inclusion of confirmatory codes, which reduced the overall number of identified events, both in PWS patients and the controls. The occurrence of arterial thrombosis events in this same group of PWS patients was highest in the 65+ year age cohort when compared to the controls. The difference in the overall rate of occurrence between the PWS population and the controls was highest across all analyses with confirmatory codes in the 65+ year age cohort.

For patients with Medicaid coverage, the largest difference in the occurrence of PE and DVT events was seen in the 65+ year age cohort. As a function of advanced age, both PWS patients and obese controls had the highest occurrence of events. While the inclusion of confirmatory codes reduced the number of these same events that were identified in the 18–64-year age cohort, there was a higher occurrence in PWS patients than their similarly-matched obese counterparts across all age cohorts. For other venous thrombotic events, there was also a high occurrence rate in the 65+ year age cohort. In both analyses, differences may have been inflated by the smaller number of PWS patients compared to the obese controls, who were almost nine times more numerous (see Table 2). The inclusion of confirmatory codes reduced the number of events in the adult study population, but there was still a higher occurrence of these events in PWS patients than the controls. The occurrence of arterial thrombosis events was higher in the 65 year + age cohort than their non-PWS counterparts. These results were influenced by whether the model included comorbidities or not. These adjustments controlled for the given limitations of the claims data, permitting an assessment of the PWS-specific contribution to thrombosis event risk. For example, while the occurrence of arterial events (i.e., stroke and heart attacks) alone was higher for the commercially-insured individuals with PWS than the individuals in the obese control group (2.2% vs. 0.5%), when controlling for comorbidities, the difference was not statistically significant (OR = 1.071, *p*-value = 0.8375) (see Figure 3). After controlling for comorbidities, commercially insured subjects appeared 2.55 times more likely than the controls to have experienced a PE or DVT event (*p*-value = 0.013) but the difference in the frequency of events between the Medicaid-insured individuals and controls was not statistically significant. The results showed a trend of a higher frequency for thrombosis events in the PWS patients than the controls, but this was not always statistically different in all analyses. In the commercial and Medicare coverage, PWS patients had a difference in PE and DVT event frequency that was statistically significant when compared to obese non-PWS patients across all age groups, while in the Medicaid coverage group, the obese non-PWS controls experienced a higher occurrence of thrombosis events overall. Due to nuances of obesity coding in claims, we suspected a higher rate of false-negative detection of obesity, as well as a selection bias toward morbidly obese patients in the obese-positive cohorts. Statistically significant risk factors were also identified in PWS patients and appeared independently in all types of insurance coverage while using the Elixhauser comorbidities index.

Overall, survival in PWS is especially threatened by obesity, which is secondary to hyperphagia from a disturbance in the hypothalamic pathways of satiety control, irregularity in hormones that regulate food intake, and reduced energy expenditure in view of poor feeding and hypotonia [14,15]. Obesity is the most common cause of metabolic complications and can reduce the quality of life. Unfortunately, obesity and its complications are major contributors of increased mortality, especially in patients with PWS when compared with the general population [16,17].

With a shortened life expectancy that is attributed to the association of the level of intellectual disability and life-threatening complications in PWS that are related to hyperphagia and obesity, our study supports the trending decrease in the number of patients within the older age groups [18]. According to Manzardo et al. [10], the survival trends for patients diagnosed with PWS is on the rise when comparing recent era (post 2000 year) versus early era (pre 2000 year) mortality trends, thus suggesting a more optimistic future for PWS. However, the long-term survival trend shows clear gender- and disease-specific impacts, as supported by our findings that associated gender and disease are potential risk factors that can be targeted to improve and increase life spans (see Table 3).

A complication of obesity, especially in adults, is circulatory problems [19]. Butler et al. [5] reported that deaths due to obesity-related factors such as cardiovascular disease and pulmonary embolism that appear in childhood and increase throughout life, as opposed to other causes of death such as gastrointestinal infections which are generally more stable, at about a 10% mortality rate, throughout life. The increase in the occurrence rates of thrombotic events in the PWS population in our study showed an increasing rate over all age cohorts, with the highest occurrence in the 65+ years age cohort. However, thrombosis has been reported as a risk factor in PWS newborns with cerebral venous thrombosis [20]. Additionally, a study by Butler et al. [21] suggested that mean C-reactive protein values, indicators of inflammation, were higher in PWS subjects but similar to those seen in the non-PWS obese individuals with cardiovascular disease. This suggests that subjects with Prader–Willi syndrome and obese non-PWS subjects are at a similarly increased risk for complications of obesity including thrombosis. However, coagulopathy in PWS has been understudied. It is important to characterize the cause of venous vs. arterial blood clots in PWS in future studies. Disturbances in *F2* (thrombin), *F5* (coagulation factor 5) and other genes such as *PROC* (protein C), *SERPINC1* (serpin family C member 1) and *PROS1* (protein S) can produce both autosomal-dominant and autosomal-recessive patterns of inheritance of blood clots in families (https://www.omim.org), and more studies are required to possibly differentiate their role in arterial and venous thromboses impacted by age of onset in both PWS patients and obese controls.

More venous thromboses (e.g., VTEs) are reported than arterial thrombosis; however, these do occur, as illustrated by a clinical report from Kusuhara et al. [22] that highlighted a 19-year-old patient with PWS with a stroke and a brain MRI that revealed abnormal signal intensities in the left basal ganglia, including the right trigone of the lateral ventricle. An angiographic examination showed the occlusion of the bilateral proximal middle cerebral arteries with basal vessels, as well an occlusion of the left vertebral artery at its origin. This suggested that an arterial thrombosis event may have occurred, but it may have also resulted from atherosclerosis that secondary to the diagnosis of type 2 diabetes.

## 5. Conclusions

Thrombotic events in the PWS population, such as PE, DVT, other venous or arterial thromboses do occur but they are rare. Regardless of the type of insurance coverage and services received, the occurrence of thrombosis events or blood clots increased with age in PWS and is a leading cause of morbidity and mortality that is further increased by obesity. Survival trends can improve in patients with PWS if the associated risk factors for thrombosis events such as age, gender, coagulopathy, cardiac arrhythmias, and, especially, obesity and its related complications are targeted for proper management early and often throughout life, as thrombotic risk factors should be deeply considered together with comorbidities.

## Figures and Tables

**Table 1:** Ten year health insurance claims dataset (2004–2014) showing distribution of patients with Prader–Willi syndrome.

Prader–Willi Syndrome	Commercial and Medicare Advantage	Medicaid
**N**	N = 1821	N = 1315
**Gender Distribution (%)**		
**Male**	N = 921 (50.6)	N = 715 (54.4)
**Female**	N = 900 (49.4)	N = 600 (45.6)
**Age Distribution (%)**		
**0–17 year**	N = 1312 (72.0)	N = 731 (55.6)
**18–64 year**	N = 464 (25.5)	N = 576 (43.8)
**65 year+**	N = 45 (2.5)	N = 8 (0.6)

**Table 2:** Unadjusted primary thrombosis event occurrence in PWS subjects on commercial/Medicare and Medicaid coverage.

**2A**	**% Experiencing Event**		**Odds Ratio (OR)**	**95% Lower Confidence Limit**	**95% Upper Confidence Limit**	***p*-Value**
**Commercial/Medicare**	**PWS Subjects**	**Controls**				
**Analysis 1**	2.10%	0.40%	4.3	2.5	7.2	<0.0001
**Analysis 2**	1.80%	0.30%	5	2.8	9	<0.0001
**Analysis 3**	3.80%	0.60%	5.3	3.5	8.1	<0.0001
**Analysis 4**	2.90%	0.50%	5.1	3.2	8.2	<0.0001
**Analysis 5**	2.20%	0.50%	3.9	2.3	6.5	<0.0001
**2B**	**% Experiencing Event**		**Odds Ratio (OR)**	**95% Lower Confidence Limit**	**95% Upper Confidence Limit**	***p*-Value**
**Medicaid**	**PWS Subjects**	**Controls**				
**Analysis 1**	3.20%	0.80%	1.9	1.2	3	0.007
**Analysis 2**	3.00%	0.70%	1.9	1.2	3.2	0.007
**Analysis 3**	5.80%	1.50%	2	1.4	2.8	0.0002
**Analysis 4**	4.80%	1.30%	1.9	1.3	2.7	0.001
**Analysis 5**	3.50%	1.50%	1.1	0.8	1.7	0.47
**Analysis 5**	2.20%	0.50%	3.9	2.3	6.5	<0.0001

Analysis 1: PE and DVT codes. Analysis 2: PE and DVT codes and confirmatory codes. Analysis 3: PE and DVT codes and other venous thrombosis codes. Analysis 4: PE and DVT codes and other venous thrombosis codes and confirmatory codes. Analysis 5: arterial thrombosis codes. Selection Criteria: subjects with > = 1 year of continuous enrollment. Diagnosis criteria: At least two PWS diagnosis (ICD-9-CM 759.81) at any time during a subject’s continuous enrollment period. *P*-values are based upon a Chi-square test.

**Table 3:** Potential significant risk factors of thrombotic events.

	Commercial/Medicare Supplement			Medicaid		
		OR	*p*-Value		OR	*p*-Value
**PE or DVT**	Age Cohort 65+ (vs. 0–17)	11.0	<0.0001	Age Cohort 65+ (vs. 0–17)	6.2	0.02
	Age Cohort 18–64 (vs. 0–17)	6.2	<0.0001	Age Cohort 18–64 (vs. 0–17)	5.2	0.0001
	Cardiac Arrhythmia	3.0	0.002	Cardiac Arrhythmia	2.8	0.0005
	Coagulopathy	4.5	0.0005	Coagulopathy	8.4	<0.0001
	Male Gender (vs. Female)	2.3	0.01	Male Gender (vs. Female)	1.0	0.9
	Obesity	3.1	0.001	Hypertension Complicated	2.6	0.003
	AIDS/HIV	13.8	0.03	Solid Tumor without Metastasis	4.0	0.0002
	Diabetes Uncomplicated	2.6	0.006	Psychosis	1.9	0.03
**PE, DVT or other thrombosis**	Age Cohort 65+ (vs. 0–17)	4.9	0.0008	Age Cohort 65+ (vs. 0–17)	9.8	<0.0001
	Age Cohort 18–64 (vs. 0–17)	4.4	<0.0001	Age Cohort 18–64 (vs. 0–17)	4.6	<0.0001
	Cardiac Arrhythmia	2.2	0.007	Cardiac Arrhythmia	2.1	0.002
	Coagulopathy	4.8	<0.0001	Coagulopathy	7.3	<0.0001
	Male Gender (vs. Female)	1.6	0.08	Male Gender (vs. Female)	1.2	0.3
	Hypertension Complicated	3.6	<0.0001	Hypertension Complicated	2.1	0.004
	Solid Tumor without Metastasis	2.1	0.05	Obesity	1.7	0.05
	AIDS/HIV	10.9	0.01	Solid Tumor without Metastasis	2.2	0.05
	Deficiency Anemia	2.4	0.009	Congestive Heart Failure	1.7	0.05
	Chronic Pulmonary Disease	1.9	0.01	Metastatic Cancer	4.7	0.009
				Rheumatoid Arthritis/Collagen	2.5	0.006

Risk factors that were identified by using stepwise logistic regression modeling are shown above as significant in relation to thrombotic events in Prader–Willi syndrome, with the top risk factors being age cohorts (65+ years or age cohort 18–64 years versus 0–17 years in both cases), coagulopathy, cardiac arrhythmias, and male gender (vs. female).
